# Supplementary material for: WordCommentsAnalyzer: A windows software tool for qualitative research
Source: F1000Res. 2018 Sep 4;7:536. Originally published 2018 May 3. [Version 2] doi: 10.12688/f1000research.14819.2 (PMC6137411; doi:10.12688/f1000research.14819.2)
Supplement: Supplementary file 3 [file f1000research-7-17614-s0002.tgz › b2a79496-4ada-47c4-9682-a99ce867836d.zip!/Eight Iranian Health Journals/Journal of Research in Rehabilitation Sciences _22.docx]

© Journal of Research in Rehabilitation Sciences . This work is licensed under CC BY-NC 4.0 (https://creativecommons.org/licenses/by-nc/4.0/).

Volume & Issue: دوره 10، شماره 3: 1393

Title: تاثیر فوری کفی کفش بر مولفه¬های فرکانسی نیروی عکس العمل زمین در فاز اتکاء دویدن

http://jrrs.mui.ac.ir/index.php/jrrs/article/view/1418

مقدمه: کفی­های کفش به­طور گسترده برای جلوگیری از صدمات مرتبط با دویدن توصیه می­شوند. با این وجود، اثرات آن­ها بر فرکانس نیروی عکس العمل زمین (GRF) هنوز مبهم است. هدف این مطالعه بررسی مقدماتی اثر 4 کفی­ کفش بر فرکانس GRF در فاز اتکاء دویدن بود.  مواد و روش­ها: 15 دانشجوی دختر (میانگین سن 85/1± 22سال، قد 71/4±162 سانتیمتر و وزن 59/5±56 کیلوگرم) انتخاب شدند. از آزمودنی­ها خواسته شد تحت شرایط کنترل (فقط کفش) و کفی (معمولی، نرم، نیمه­سخت، سخت) روی صفحه­ی نیروسنج بدوند. GRF  عمودی و قدامی-خلفی در تابع فرکانس با استفاده از تبدیل فوریر محاسبه شد. برای آزمون فرضیات از تحلیل واریانس (آنوا) یک عامله با اندازه گیری مکرر استفاده شد (05/0≥p). یافته­ها: نتایج آماری نشان داد اختلاف معنی­داری بین کفی­ها در فرکانس با توان 5/99%  GRF عمودی (001/0P=) و قدامی-خلفی (049/0P=) و میانه فرکانس GRF قدامی- خلفی (012/0P=) وجود داشت. نتیجه گیری: افزایش سختی کفی­ها، میزان تواتر با توان 5/99% GRF عمودی و قدامی- خلفی و میانه تواتر GRF قدامی- خلفی را کاهش داد. بر این اساس، فرکانس با توان 5/99% GRF عمودی و قدامی-خلفی و میانه فرکانس GRF قدامی-خلفی می­توانند نشان دهنده اثرات کفی­های کفش بر پا در طی دویدن باشند.

Title: بررسي روایی آزمون ارزیابی عملکردی مهارت های خود مراقبتی در بررسی خود مراقبتی بيماران مولتیپل اسکلروزیس بزرگسال مراجعه كننده به بخش‌هاي توانبخشي شهر تهران

http://jrrs.mui.ac.ir/index.php/jrrs/article/view/1235

مقدمه: فعاليت‌هاي خود مراقبتی به بخشي از فعاليتها اطلاق مي‌شود كه فرد به منظور حفظ خويشتن و كسب استقلال در زندگي روزمره‌اش انجام مي‌دهد. يكي از آزمون‌هاي جامع در زمينه ارزيابي فعاليت های خود مراقبتی آزمون ارزیابی عملکردی مهارت های خود مراقبتی است. هدف از مطالعه حاضر، بررسي روایی آزمون ارزیابی عملکردی مهارت های خود مراقبتی، در بيماران مبتلا به مولتیپل اسکلروزیس است.  مواد و روش ها: تحقیق حاضر یک مطالعه غیر تجربی از نوع روش شناختی است، که طی آن آزمون ارزیابی عملکردی مهارت های خود مراقبتی به زبان فارسی ترجمه و معادل سازی شد. در این پژوهش روایی صوری و روایی محتوا بررسی گردید. جهت بررسي همگن بودن نظرات كارشناسان در روایی محتوا از آزمون كروسكال واليس استفاده گردید. در روایی صوری جهت تهیه نسخه نهایی 30 نفر از افراد بزرگسال مبتلا به مولتیپل اسکلروزیس مشارکت نمودند.  یافته ها: ميانگين نمرات كارشناسان و درصد روايي در زمينه فعاليت هاي روزمره زندگي در بعد استقلال 93/3 و روايي 98 درصد، در بعد ايمني 80/3 و روايي 95 درصد و در بعد نتيجه 93/3 و روايي 98 درصد مي‌با‌شد. درصد روايي در زمينه استفاده ابزار در فعاليت های روزمره زندگي در بعد استقلال 77/3 و روايي 94 درصد، در بعد ايمني 70/3 و روايي 92 درصد و در بعد نتيجه 84/3 و روايي 96 درصد مي‌با‌شد. این درصد در زمينه تحرك عملكردي در بعد استقلال 40/3 و روايي 85 درصد، در بعد ايمني 40/3 و روايي 85 درصد و در بعد نتيجه 36/3 و روايي 84 درصد مي باشد. و در نهایت، درصد روايي در زمينه خود مراقبتی (كل آزمون) در بعد استقلال 72 /3 و روايي 93 درصد، در بعد ايمني 65/3 و روايي 91 درصد و در بعد نتيجه 75/3 و روايي 94 درصد مي باشد.  نتیجه گیری: این یافته ها نشان داد که آزمون ارزیابی عملکردی مهارت های خود مراقبتی، در هر سه بعد استقلال، ‌ايمني و نتيجه داراي روایی بسيار بالايي است و می توان آن را به عنوان ابزاری روا به منظور بررسی خود مراقبتی بيماران مولتیپل اسکلروزیس بزرگسال به کار برد.

Title: مطالعه تاثیر تصحیح پروناسیون بیش از حد مفصل ساب تالار با استفاده از روش Taping قوس داخلی پا بر راستا و درد زانو

http://jrrs.mui.ac.ir/index.php/jrrs/article/view/1427

مقدمه:  در بررسی عوامل تأثیر گذار بر بیومکانیک مفصل زانو، مشکلات پا به دلیل برهم زدن راستای نیروها بسیار مورد توجه هستند . از این رو هدف این مطالعه تعيين ارتباط بين پروناسيون بيش از حد در مفصل ساب تالار و ميزان زاويه Q در زانو و تعيين تاثير Taping قوس داخلي پا بر تغييرات آنها است.   مواد و روش ها: مطالعه بر روی افراد داوطلب مراجعه کننده به کلینیک فیزیوتراپی دانشکده علوم توانبخشی شهر شیراز و در محدوده سنی 40- 20 سال انجام گرفت. افراد تحت مطالعه در دوگروه جای داده شدند :  گروه اول (PSK = Pronated Symptomatic Knee) : شامل 38فرد داراي پروناسيون بيش از حد در مفصل ساب تالار و علائم درد در مفصل زانو بود.  گروه دوم (PASK = Pronated Asymptomatic Knee): شامل 38 فرد داراي پروناسيون بيش از حد در مفصل ساب تالار که در مفصل زانو بدون علائم درد بودند. در اين دو گروه زواياي Q و ساب تالار قبل و بعد از انجام Taping قوس داخلي پا بوسیله گونیامتر اندازه گيري شد. برای تجزیه و تحلیل آماری از آزمون های آماری پیرسون،اسپیرمن، تی زوجی و ویلکاکسون استفاده شد.   یافته ها: بين پروناسيون بيش از حد در مفصل ساب تالار و ميزان زاويه Q در هر دو گروه PSK , PASK قبل از انجام Taping، همبستگي معني داري وجود داشت.(در زنان گروه PSK و PASK به ترتیب:002/0 p= و001/0 p= و در مردان PSK و PASK به ترتیب:001/0 p= و001/0 p=) روش Taping در گروه هاي PSK و PASK، بر کاهش ميزان زاويه ساب تالار و ميزان زاويه Q تاثير گذار بود.   نتیجه گیری: به نظر می رسد در افراد داراي پروناسيون بيش از حد، به کار بردن روش Taping قوس داخلی پا باعث کاهش زاویه Q و در نتیجه بهبود بيومكانيك مفصل زانو می گردد.

Title: عناصر درونی و عوامل بیرونی موثر بر آموزش بالینی از دیدگاه دانشجویان فیزیوتراپی: مطالعه کیفی

http://jrrs.mui.ac.ir/index.php/jrrs/article/view/1677

مقدمه: آموزش بالینی بخش جدایی­ناپذیر آموزش فیزیوتراپی بوده و از جایگاه ویژه­ای برخوردار است. طی این دوره، دانشجویان به تدریج کسب تجربه نموده و ذهنشان را برای حل مشکل بیمار آماده می­سازند. آموزش بالینی مانند تمام برنامه­ها از فرایندها و عناصری تشکیل شده که برخی عوامل بیرونی نیز بر آنها اثر می­گذارند. این پژوهش توجه خود را به عناصر درونی و عوامل بیرونی موثر بر آموزش بالینی بر مبنای تجربیات دانشجویان معطوف کرده است. روش­ها: در این مطالعة کیفی از روش تحلیل محتوا استفاده شد. جامعة پژوهشی شامل دانشجویان سال آخر کارشناسی فیزیوتراپی بوده که تعداد 21 نفر به صورت هدفمند انتخاب گردیدند. از بحث گروهی متمرکز جهت گردآوری داده­ها استفاده شد. یافته­ها: پس از تجزیه و تحلیل داده­ها و ترکیب کدها، 11 زیرمضمون، چهار مضمون ظهور یافتند. مضمون­های عناصر «انسانی» و «فیزیکی» حیطه عناصر درونی و مضمون­های «سیاست­های دانشگاه» و «رویکردها و رویه­ها» حیطة عوامل بیرونی را تشکیل می­دهند. عدم همسویی برخی سیاست­ها و رویکردهای موجود با اهداف دورة آموزش بالینی، موجب عدم غنای تجربة بالینی می­گردد. نتیجه­گیری: با توجه به کدهای به دست آمده، عناصر انسانی جایگاه برجسته­تری در مقایسه با عناصر فیزیکی داشتند. هرچند مداخله در برخی عناصر مانند ویژگی­های بیمار و ویژگی­های شخصیتی استاد و کارآموز در توان گروه­های آموزشی نیست اما شناسایی آنها به عنوان عناصر اثرگذار می­تواند تجربه­ای متفاوت را برای کارآموزان رقم بزند. همچنین به نظر می­رسد مواردی چون نظارت دقیق نظام آموزشی دانشگاه بر عملکرد استادان و دانشجویان، تعدیل سطح پذیرش دانشجویان و نسبت­های جنسیتی و نیز افزایش سقف واحدها می­تواند راهگشا باشد.

Title: بررسي تاثير استفاده از فعاليت هدفمند باغباني بر کیفیت زندگی دانشجويان با درجه افسردگی متوسط تا عمیق ساكن در خوابگاه گلستان دختران دانشگاه علوم پزشكي جندي شاپور اهواز

http://jrrs.mui.ac.ir/index.php/jrrs/article/view/1376

مقدمه: دانشجویان با عوامل استرس زای بسیاری در دوران تحصیل مواجه هستند. استرس می تواند منجر به بروز بیماری های جسمی و روانی، اختلال در عملکرد و پایین آمدن کیفیت زندگی دانشجویان گردد. یکی از مداخلات بر مبنای طبیعت، استفاده از فعالیت هدفمند باغبانی می باشد. این مطالعه با هدف بررسی تاثیر استفاده از فعالیت هدفمند باغبانی روی کیفیت زندگی دانشجویان با درجه افسردگی متوسط تا عمیق ساکن در خوابگاه دختران علوم پزشکی جندی شاپور اهواز انجام شده است. مواد و روشها: این مطالعه از نوع کارآزمایی بالینی گواه دار تصادفی بود و در سال تحصیلی 91-1390 انجام شد. نمونه مورد مطالعه 50 نفر از دانشجویان افسرده ساکن در خوابگاه دختران گلستان علوم پزشکی جندی شاپور اهواز بود. دانشجویان افسرده به صورت تصادفی در دو گروه آزمایش وگواه قرار گرفتند. دو گروه در پیش آزمون کیفیت زندگی BREF WHOQOL- شرکت کردند. سپس جلسات باغبانی به مدت 3 جلسه در هفته به مدت 2 ماه در محیط حیاط خوابگاه گلستان برای گروه آزمایش انجام گرفت. دو گروه پس از پایان مداخله مجددا با همان آزمون مورد ارزیابی قرار گرفتند. نتایج به کمک نرم افزار SPSS18 مورد بررسی آماری قرار گرفت. نتایج: بررسی آماری به کمک آزمونهای لوین، انکوا و مانکوا نشان داد که کیفیت زندگی کلی در گروه آزمایش پس از مداخله درمانی افزایش یافته است. همچنین در حیطه های کیفیت زندگی در زمينه سلامت جسمانی وسلامت محیط در گروه آزمایش بهبود وجود داشته است  و در حیطه های کیفیت زندگی در زمینه سلامت روانی و سلامت روابط اجتماعی در گروه آزمایش تغییر معناداری وجود نداشته است. بحث: کیفیت زندگی به دنبال استفاده از فعالیت هدفمند باغبانی در دانشجویان افسرده ساکن در خوابگاه دختران افزایش می یابد. تغییر در حیطه های مختلف زیرمجموعه کیفیت زندگی به دنبال استفاده از فعالیت هدفمند باغبانی در دانشجویان افسرده نیازمند بررسی های بیشتر می باشد.

Title: بررسی تاثیر حداکثر اکسیژن مصرفی بر شاخص¬های پويايي اكسيژن دوره ريكاوري پس از فعاليت بيشينه پیوسته در زنان بسکتبالیست

http://jrrs.mui.ac.ir/index.php/jrrs/article/view/439

مقدمه: دوره ريكاوري، دوره جدايي ناپذير پس از ورزش است كه در این دوره ميزان متابوليسم بالاتر از سطوح استراحتي است. درک چگونگي پويايي اكسيژن مصرفي در دوره ريكاوري با استفاده از شاخصه هاي آن، پس از فعاليت ورزشی می تواند به مربيان، ورزشكاران و بیماران با درجات مختلف نارسایی های قلبی در طراحي برنامه تمريني كمك کند. بر همين اساس هدف تحقیق حاضر بررسی تاثیر حداکثر اكسيژن مصرفی بر شاخص های پویایی اکسیژن دوره ريكاوري پس از فعاليت بيشينه پيوسته در زنان بسکتبالیست می باشد. مواد و روش ها: برای انجام این مطالعه، 7 زن بسکتبالیست نخبه با میانگین سنی 5/1 ±21 سال، وزن 7/5±71/60کیلوگرم، قد 6/5±3/168 سانتی متر، شاخص توده بدنی 7/1 ±7/21 و حداکثر اکسیژن مصرفی 4/3 ± 4/41 میلی لیتر بر کیلوگرم در دقیقه به صورت نمونه گیری هدفمند و در دسترس انتخاب شدند. برای جمع آوری داده های شاخص های پویایی اکسیژن دوره ريكاوري از دستگاه سنجش گازهای تنفسی (GAS ANALYZER) استفاده شد. برای تجزیه و تحلیل داده ها از آزمون Paired t در سطح معنی­داری 005/0= P استفاده گردید. نتایج: پس از فعالیت بیشینه پیوسته، در نصف زمان ریکاوری اکسیژن (005/0= P) و نصف زمان ریکاوری نبض اکسیژن (001/0= P) تفاوت معنی­داری مشاهده شد. بحث: آزمودنی هایی که حداکثر اكسيژن مصرفی بالاتری دارند، پویایی اکسیژن سریع تر و نصف زمان ریکاوری کوتاه تری داشته و بازگشت به حالت اولیه در آنان سریع تر است.

Title: ارتباط قدرت مرکزی بدن با تعادل ایستا و پویا در ورزشکاران مرد اسکی اسنوبرد

http://jrrs.mui.ac.ir/index.php/jrrs/article/view/1527

مقدمه: اسکی، ورزشی با سرعت بالا است که خطر سقوط و افتادن­ در آن رایج است. هدف از مطالعه حاضر، بررسی ارتباط قدرت ناحیه مرکزی بدن اسنوبردبازان با تعادل ایستا و پویا بوده است. مواد و روش­ها: نمونه آماری تحقیق حاضر شامل 40 نفر­ از ورزشکاران مرد (میانگین سن 30/3±85/26سال، قد 08/6±45/180سانتی­متر، وزن 14/6±86/79کیلوگرم و سابقه ورزشی 53/4±70/12فصل) با حداقل 6 فصل سابقه­ی فعالیت در اسکی اسنوبرد بودند. آزمودنی­ها به روش نمونه گیری در دسترس انتخاب شدند. در این پژوهش، قدرت مرکزی بدن شامل آزمون­های قدرت ایزومتریک ابداکشن هیپ و قدرت ایزومتریک چرخش خارجی هیپ­ بوده است که به وسیله ابزار دینامومتر دستی نیکولاس اندازه­گیری شد. برای ارزیابی تعادل ایستا و پویا نیز به ترتیب از آزمون­های ­عملکردی لک­لک و ستاره استفاده شده است. از نرم افزار 18SPSS تحت ویندوز ویستا برای تجزیه و تحلیل اطلاعات استفاده شده است. داده­ها پس از بررسی اطلاعات شاخص­های مرکزی و پراکندگی، با استفاده از آزمون همبستگی پیرسون مورد تجزیه و تحلیل آماری قرار گرفتند (05/0P˂). یافته­ها: یافته­ها نشان دادند که بین قدرت مرکزی بدن با تعادل ایستا (001/0P=) و تعادل پویا (001/0P=) در اسنوبردبازان ارتباط معنی­داری وجود دارد. نتيجه­گيری: با توجه به ارتباط معنی­دار میان قدرت مرکزی بدن و تعادل ایستا و پویای ورزشکاران اسکی اسنوبرد در این تحقیق و اهمیت حفظ تعادل در این رشته ورزشی، تقویت عضلات مرکزی برای بهبود تعادل اسنوبردبازان جهت پیشگیری از وقوع آسیب­های ناشی از ضعف تعادل و در نتیجه بهبود عملکرد این ورزشکاران در کنار دیگر برنامه­های تمرینی ضروری به نظر می­رسد.

Title: ارزیابی تأثیر تمرین حفظ تعادل بر روی بهبود پایداری بیماران مولتيپل‌اسكلروزيس با استفاده تحلیل غیرخطی سمپل‌انتروپی

http://jrrs.mui.ac.ir/index.php/jrrs/article/view/1325

مقدمه: از بین رفتن غلاف میلین در بیماران مولتيپل‌اسكلروزيس (MS) باعث بروز مشکل در انتقال پیام‌های عصبی می‌شود و در نتیجه موجب اختلال در سیستم عصبی-عضلانی ازجمله اختلال در حفظ تعادل شخص بیمار می‌گردد. اندازه‌گیری و ارزیابی نوسانات مرکز ثقل بدن در طی فعالیت‌های گوناگون روش کارآمدی جهت بررسی سیستم عصبی-عضلانی و حفظ تعادل شخص می‌باشد چرا که تغییرات پیچیدگی نشانه‌ای از تولید اطلاعات از نوسانات وضعی است. این امر با کمک معیار انتروپی امکان پذیر می‌باشد و می‌توان با استفاده از آن میزان پیچیدگی سینماتیک بدن را بررسی کرد. مواد و روش‌ها: در این بررسی سیگنال تغییرات مرکز ثقل بدن، از 20 شخص مبتلا به بیماری ام.اس، پیش از 24 جلسه تمرینات حفظ تعادل و پس از آن ثبت شد. این تغییرات با دو روش تحلیل انتروپی مورد ارزیابی قرار گرفت. یافته‌ها: پس از 12 هفته انجام تمرینات، نتایج هر دو روش تحلیل، افزایش معنادار میزان انتروپی محاسبه شده از نوسانات مرکز ثقل بیماران را نشان داد (افزایش پیچیدگی در سیگنال ثبت شده). این افزایش برای حفظ تعادل در صفحه داخلی-خارجی (فرونتال) بیشتر از صفحه قدامی-خلفی (ساژیتال) بود (به ترتیب با 910/2- t=، 009/0=Pvalue و 331/2-=t و 031/0= Pvalue) نتیجه‌گیری: افزایش مقدار انتروپی بدست آمده، نشان‌دهنده تأثیر تمرینات بر روی بهبود تعادل در بیماران می‌باشد. در نتیجه، این نوع تمرینات به عنوان یک روش مداخله تأثیرگذار برای بهبود تعادل بیماران ام.اس پیشنهاد می‌شود.

Title: مقایسه اختلاف طول اندام تحتانی و استقامت عضله مربع کمری در زنان مبتلا به کمر درد مزمن و زنان سالم

http://jrrs.mui.ac.ir/index.php/jrrs/article/view/981

مقدمه: اختلاف طول اندام تحتانی و ضعف عضله مربع کمری می توانند از عوامل مرتبط با کمر درد محسوب شوند که در بسیاری از موارد در طی معاینات بالینی نادیده گرفته می شوند.هدف از این پژوهش مقایسه اختلاف طول اندام تحتانی و ضعف عضله مربع کمری در گروه زنان مبتلا به کمردرد مزمن و زنان سالم بوده است. مواد و روش ها: در يك مطالعه مورد- شاهد، چهل و نه زن بیمار مبتلا به کمردرد مزمن با چهل و نه زن سالم مقایسه شدند. برای بررسی اختلاف طول اندام تحتانی از دو روش اندازه گیری اختلاف طول ظاهری و طول حقیقی استفاده شد. برای محاسبه استقامت عضله مربع کمری از آزمون side bridge استفاده گردید. بررسی آماری توسط نرم افزار SPSS ویرایش 15 با استفاده از آزمون تی- تست انجام شد(05/0=P ). یافته ها: از نظر اختلاف طول اندام تحتانی در هر دو روش اختلاف معنی داری در دو گروه مشاهده نشد(828/0=p ) و(356/0=p). از نظر استقامت عضله مربع کمری اختلاف معنی داری بین دو گروه دیده شد. استقامت عضلات مربع کمری سمت راست و چپ بین دو گروه اختلاف معنی داری را نشان داد (به ترتیب 002/0=p و001/0=p). نتیجه گیری: در این تحقیق مشخص گردید که نمی توان رابطه ای بین اختلاف طول اندام تحتانی و کمردرد در نظر گرفت، كه ممكن است به دليل تطابق فرد با اختلاف طول اندام باشد. همچنین در بیماران مبتلا به کمردرد مزمن کاهش استقامت عضله مربع کمری دیده شدكه اين مسئله نشان دهنده نقش موثر اين عضله در ايجاد ثبات و حمايت از مهره هاي كمر مي باشد. هرچند که در این زمینه نیاز به مطالعات بیشتری است.

Title: سرگیجه در سالمندان

http://jrrs.mui.ac.ir/index.php/jrrs/article/view/1568

مقدمه: سرگیجه، عدم تعادل و زمین خوردن ناشی از آن در سالمندان، یکی از اصلی ترین چالش های پیش روی بیمار و متخصصین بوده و موارد متعددی، شناسایی و درمان سرگیجه در سالمندان را در مقایسه با سایر گروه های بزرگسالان با دشواری رو به رو می سازد. بی توجهی به علل سرگیجه های سالمندی نه تنها تهدیدی بر زندگی و سلامت بیمار و عامل آسیبهای جسمی-روحی متعدد است، بلکه میتواند منجر به انزوای فرد از محیط های اجتماعی شود. در این مطالعۀ مروری قصد داریم به بررسی نکات کلیدی در سرگیجۀ سالمندان بپردازیم. مواد و روشها: در این پژوهش مروری، پایگاه های استنادی Pubmed, Google Scholar, Cochrane,  و Science Direct با کلید واژه های Geriatrics, Elderly, Older people, Imbalance, Disequilibrium, Vertigo در فاصلۀ سالهای 1975 تا 2013 مورد بررسی قرار گرفت. یافته ها:  در برسی ها تعداد 218 مقاله پژوهشی و سی مقاله مروری به دست آمد. پس از بررسی عنوان، مقدمه و بحث در این مقالات،  تعداد 45 مقاله با محتوای مورد نظر به طور کامل بررسی شد. نتیجه گیری: رشد اخیر جمعیت سالمند همراه با افزایش ناتوانی های جسمی ، شیوع سرگیجه و اختلالات تعادلی در این گروه سنی را افزایش داده است. گیجی به اولین عامل مراجعه سالمندان 75 ساله به مراکز درمانی تشخیص داده شده و 80% از سالمندان بالای 75 سال، از سرگیجه و عدم تعادل شکایت دارند. بسیاری از اختلالات مرکزی و عروقی شایع در سالمندان تظاهراتی مشابه با سرگیجه های خوشخیم محیطی دارند که میتواند روند تشخیص را با دشواری روبرو سازد. بعلاوه درمانهای توانبخشی در این گروه سنی به دلیل همراه بودن ِ آسیب های عصب شناختی و ضعف عضلانی با تاخیر همراه است. تظاهرات بالینی و ویژگی های نیستاگموس های تولیدی در بیمار سالمند از کلیدی ترین یافته ها جهت افتراق سرگیجه های محیطی و مرکزی هستند.
